# Supplementary material for: VirBR, a transcription regulator, promotes IncX3 plasmid transmission, and persistence of blaNDM-5 in zoonotic bacteria
Source: Nat Commun. 2024 Jun 28;15:5498. doi: 10.1038/s41467-024-49800-1 (PMC11214620; doi:10.1038/s41467-024-49800-1)

Supplementary data for: VirBR, a transcription regulator, promotes IncX3 plasmid transmission, and persistence of blaNDM-5 in zoonotic bacteria

Tengfei Ma[1#], Ning Xie[1#], Yuan Gao[1], Jiani Fu[1], Chun E Tan[1], Qiu E Yang[2], Shaolin Wang[1], Zhangqi Shen[1], Quanjiang Ji[3], Julian Parkhill[4], Congming Wu[1], Yang Wang[1*], Timothy R. Walsh[5*], Jianzhong Shen[1*]

Supplement Table 1 the Up-regulated genes

Gene	Location	Annotation/Production
virBR	IncX3 plasmid	T4SS related transcription regulator factor
actX	IncX3 plasmid	*nusG* family gene
virB1	IncX3 plasmid	T4SS related gene
virB2	IncX3 plasmid	T4SS related gene
virB3/4	IncX3 plasmid	T4SS related gene
virB5	IncX3 plasmid	T4SS related gene
virB6	IncX3 plasmid	T4SS related gene
virB8	IncX3 plasmid	T4SS related gene
virB9	IncX3 plasmid	T4SS related gene
virB10	IncX3 plasmid	T4SS related gene
virB11	IncX3 plasmid	T4SS related gene
virD4	IncX3 plasmid	T4SS related gene
taxB	IncX3 plasmid	T4SS related gene
trbM	IncX3 plasmid	T4SS related gene
eeX	IncX3 plasmid	EexN family lipoprotein
parB	IncX3 plasmid	Partitioning protein
hflB	IncX3 plasmid	ATP-dependent zinc metalloprotease
topB	IncX3 plasmid	DNA topoisomerase III
h-ns	IncX3 plasmid	DNA-binding transcriptional regulator
hp_3	IncX3 plasmid	ypothetical protein
hp_4	IncX3 plasmid	ypothetical protein
hp_5	IncX3 plasmid	T4SS related gene
hp_7	IncX3 plasmid	ypothetical protein
hp_6	IncX3 plasmid	ypothetical protein
sulA	Chromosome	SOS-Response related gene
lacZ	Chromosome	Aalacotsidase
yebG	Chromosome	SOS-Response related gene
dinD	Chromosome	DNA-damage-inducible protein
hp	Chromosome	ypothetical protein
lexAR	Chromosome	SOS-Response related gene
hp	Chromosome	ypothetical protein
fdhF_2	Chromosome	Formate dehydrogenase H
ahpF	Chromosome	Alkyl hydroperoxide reductase subunit F
recA	Chromosome	SOS-Response related gene

hp	Chromosome	ypothetical protein
dinB	Other plasmids	SOS-Response related gene
dinI	Other plasmids	SOS-Response related gene
lexA	Other plasmids	SOS-Response related gene

Supplement Table 2 the Down-regulated genes

Gene	Location	Annotation/Production
cysA	Chromosome	ABC transporter ATP binding subunit
asnB	Chromosome	Asparagine synthetase B
hp	Chromosome	ypothetical protein
hp	Chromosome	Hypothetical protein

Supplement Table 3 Bacteria/plasmids used in this study

Bacteria/plasmids	Description	References
3R	*E. coli* isolated from a chicken cloaca sample from a farm in Qingdao in 2015	[1]
8R	*E. coli* isolated from a chicken cloaca sample from a farm in Qingdao in 2015	[1]
K-12 BW25113	Δ(*araD-araB*)*567*, Δ*lacZ4787* (::*rrnB-3*), λ-, *rph-1*, Δ(*rhaD-rhaB*)*568*, *hsdR514*	[2]
BL21(DE3)	F-*ompT hsdSB*(rB- mB-) gal dcm λ(DE3) Ω PtacUV5::T7 polymerase	Novagen
J53	Sodium azide resistant recipient strain	[3]
pUC19-Apr	pUC19 carrying *aac(3)*-IVa gene	This study
pACYC184	Cm^R	NEB
pGFP	GFP reporter plasmid; APR^R	This study
pKD3	Cm^R	[2]
pKD46-APR	pKD46 carrying *aac(3)*-IVa gene	This study
pCP20-Spe	pCP20 carrying *aadA* gene	This study
pUC19 *virBR*	pUC19 carrying *virBR* with native promoter	This study
pACYC194 *virBR*	pACYC194 carrying *virBR* with native promoter	This study
pGFP-*taxC*	pGFP carrying 5'untranslated region of *taxC* gene	This study
pGFP-*actX*	pGFP carrying 5'untranslated region of *actX* gene	This study
pGFP-*virB1*	pGFP carrying 5'untranslated region of *virB1* gene	This study
pGFP-*taxC-virBR*	pGFP carrying 5'untranslated region of *taxC* gene and *virBR* with native promoter	This study
pGFP-*actX-virBR*	pGFP carrying 5'untranslated region of *actX* gene and *virBR* with native promoter	This study

pGFP-*virB1*-*virBR*	pGFP carrying 5'untranslated region of *virB1* gene and *virBR* with native promoter	This study
pET28a	*oriV*pBR322; *P*T7; expression vector (Kan^R)	Novagen
IncX3 Δ*virBR*	Deletion *virBR* in plasmid p3R-4	This study
IncX3 Δ*virB1/2*	Deletion *virB1/2* in plasmid p3R-4	This study
IncX3 Δ*actX*	Deletion *actX* in plasmid p3R-4	This study

Ap, ampicillin; Cm, chloramphenicol; APR, apramycin; Sp, Spectinomycin

Supplement Table 4 Primer used in this study

Primers	Sequence	References
virBR-del-F	GCCCTGGAAAAGAAAGAAACAAAAGCACTGATAGAAAAACAG GAGGTGCTgtgtaggctggagctgcttcg	
virBR-del-R	AAAGCACCTCCCGTAAACGGGAGGGCTTCGGCGATTCAGGAAC GGGAATTcatatgaatatcctccttag	
virBR-F	CGTCCGGTTTTTCCCTTGATG	
virBR-R	CGACCGGAGACAACGGATT	
pUC-*virBR*-1F	ggcagtgagcgcaacgcaatTTTAATGAGTGTGGTTTAAGTAAATCTGA	
pUC-*virBR*-1R	ctTTTATATTCCTCGCGAGGGAAT	
pUC-*virBR*-2F	cctcgcgaggaatataaaAGAAAAACAGGAGGTGCTATGAAAA	
pUC-*virBR*-2R	ttgatgcctggagatccttaTTATTCTGCCTCTGGTGTGTCG	
pUC19-v-F	TAAGGATCTCCAGGCATCAAATAA	
pUC19-v-R	ATTGCGTTGCGCTCACTGC	
pAC-*virBR*-F	gtcagccccatacgatataaTTTAATGAGTGTGGTTTAAGTAAATCTGA	
pAC-*virBR*-R	cctcccagagcctgataaaaTATAAACGCAGAAAGGCCCACC	
pACYC184-v-F	TTATATCGTATGGGGCTGACTTCA	
pACYC184-v-R	TTTTATCAGGCTCTGGGAGGC	This study
actX-p-F	gggcctttctgcgtttataTTCATACTCTCCGGTAACTTATTGATTA	
actX-p-R	agttcttctcctttactcatAGTTTTTCCTTTCAGGCGCTC	
virBR-g-F	atatttctagatttcagtgcTTTAATGAGTGTGGTTTAAGTAAATCTGA	
virBR-g-R	gggcctttctgcgtttataTTCATACTCTCCGGTAACTTATTGATTA	
taxA-p-F	atatttctagatttcagtgcAATTCCCGTTCCTGAATCGC	
taxA-p-R	agttcttctcctttactcatTTTGCGGCGTTTGCCCTA	
virB1-p-F	atatttctagatttcagtgcTACACTCTACCTGCCCTCAGCTT	
virB1-p-R	agttcttctcctttactcatGATTTTCTCCCGGCCAGAAC	
pET28a-V-F	CATATGGCTGCCGCGCGGCA	
pET28a-V-R	CTCGAGCACCACCACCACCA	
virBR-protein-F	gtgccgcgcggcagccatatgATGAAAAAACAGGAGTTTTTAGATTTTATC	
virBR-protein-R	gtggtggtggtggtgctcgagTTATTCTGCCTCTGGTGTGTCG	
VirB1-RT-F	TGCCGAGATAATACCGAAGG	
VirB1-RT-R	ATTTTCGCAGGGGTCAAAC	
VirB2-RT-F	GCTGCATTGATGCTTTGTG	
VirB2-RT-R	ATGCTGGCGTGAGAAGATAG	

VirB3/4-RT-F	ATCTCTCCCGGTTTCATTCC
VirB3/4-RT-R	TGGTCAGAGGCAGTGATTTG
VirB5-RT-F	TAAGGGGCTGGGATATACGG
VirB5-RT-R	TGCCAGAGATACAGCGTTTG
VirB6-RT-F	GCAATTAAAGCGGGAATGAC
VirB6-RT-R	GCTGGGAAGCATAGGTTTTG
VirB7/8-RT-F	GAATTGCCGTAGGTGTGTTG
VirB7/8-RT-R	GCGCTTCATTTTCAGAGAGG
VirB9-RT-F	CTCTTAACAAGCGGCAGGAG
VirB9-RT-R	TCGCCGCTCATAACAAAGAC
VirB10-RT-F	TCCTCAAATTCCGGTGGTAG
VirB10-RT-R	TGTCCAGCGAGCACTTAATC
VirB11-RT-F	CGGCCTGATGAACTGTTTG
VirB11-RT-R	ATAGACACCGTTTCCCGTTC
VirD4-RT-F	CTCTTTATGGTGACGCGAAG
VirD4-RT-R	TATTGCAGCCCCTTTACCTG
R20A-F	aacagaggcggggagctgtcGCGTTTTCGCTGGGATTTAA
R20E-F	aacagaggcggggagctgtcGAATTTTCGCTGGGATTTAA
R20-R	AGGGCATGGAAGGCGACA
R42A-F	ggactaacgaggccgggttaGCGGTCTGGAGTATACTAAGCGGTAACA
R42E-F	ggactaacgaggccgggttaGAAGTCTGGAGTATACTAAGCGGTAACA
R42-R	tgtgtcgccttccatgccctGCCGGGCATCATGGAGCC
R67A-F	gggaaagaatgagtaacctcGCGCGCTGGCTCCATGAT
R67E-F	gggaaagaatgagtaacctcGAACGCTGGCTCCATGAT
R67-R	aacgaaaggcccagtctttcGACTGAGCCTTTCGTTTTATTTGA

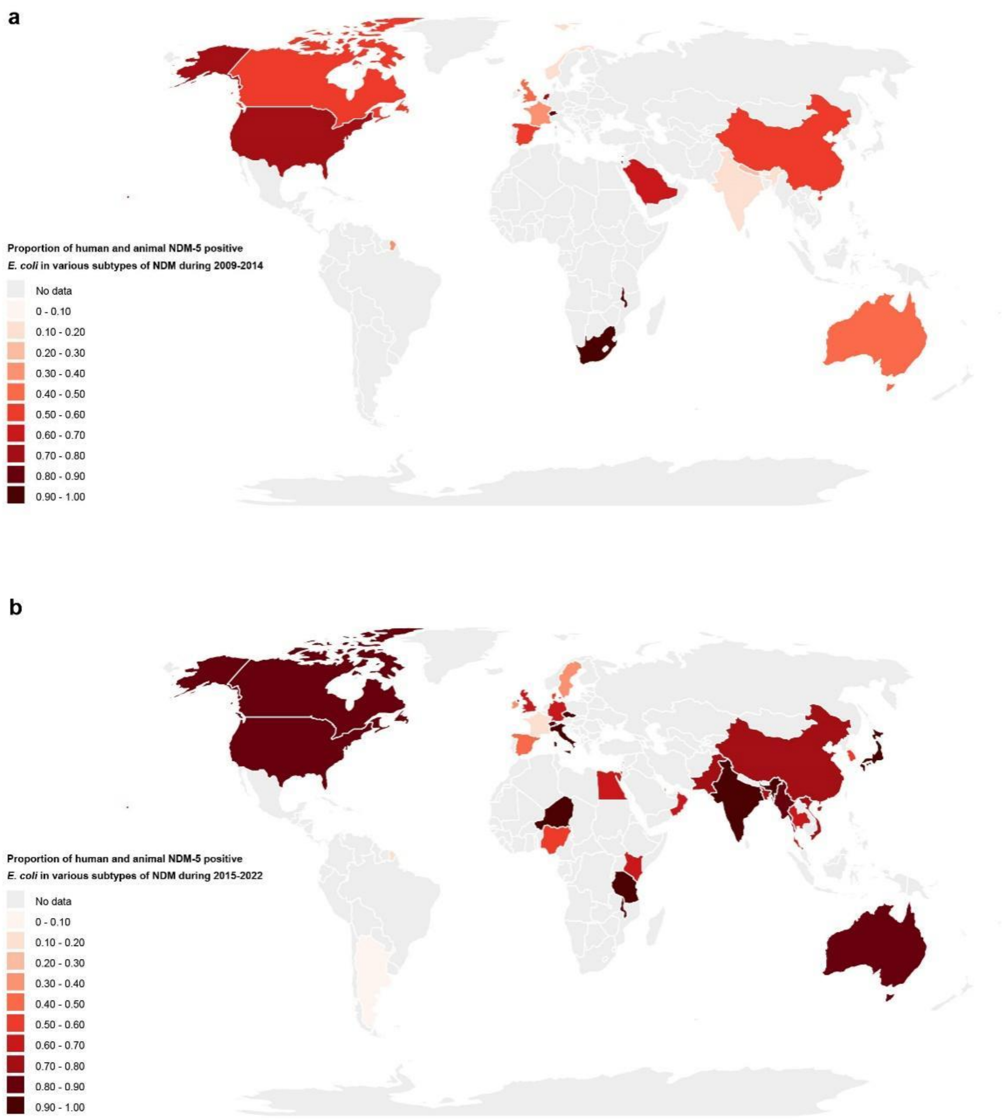

Fig. S1 The proportion of *bla*$_{\text{NDM-5}}$ of NDM-producing *E. coli* from human and animal in 2009-2014 and 2015-2022. a. The proportion of *bla*$_{\text{NDM-5}}$ of NDM-producing *E. coli* from human and animal in 2009-2014. **b.** The proportion of *bla*$_{\text{NDM-5}}$ of NDM-producing *E. coli* from human and animal in 2015-2022.

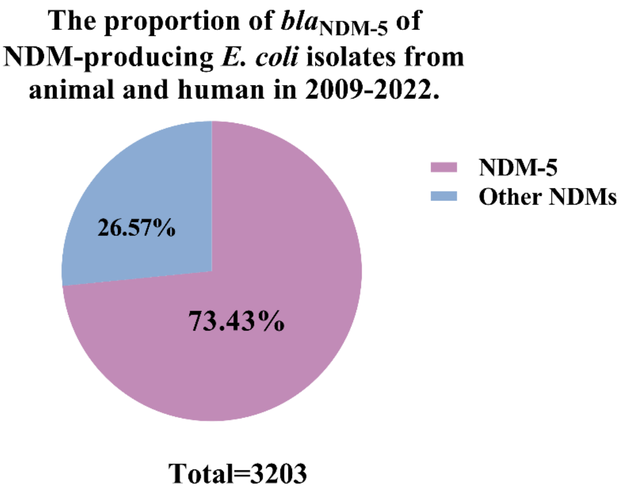

Fig. S2 The proportion of *bla*$_{\mathrm{NDM-5}}$ of NDM-producing *E. coli* from animal and human in the world in 2009-2022.

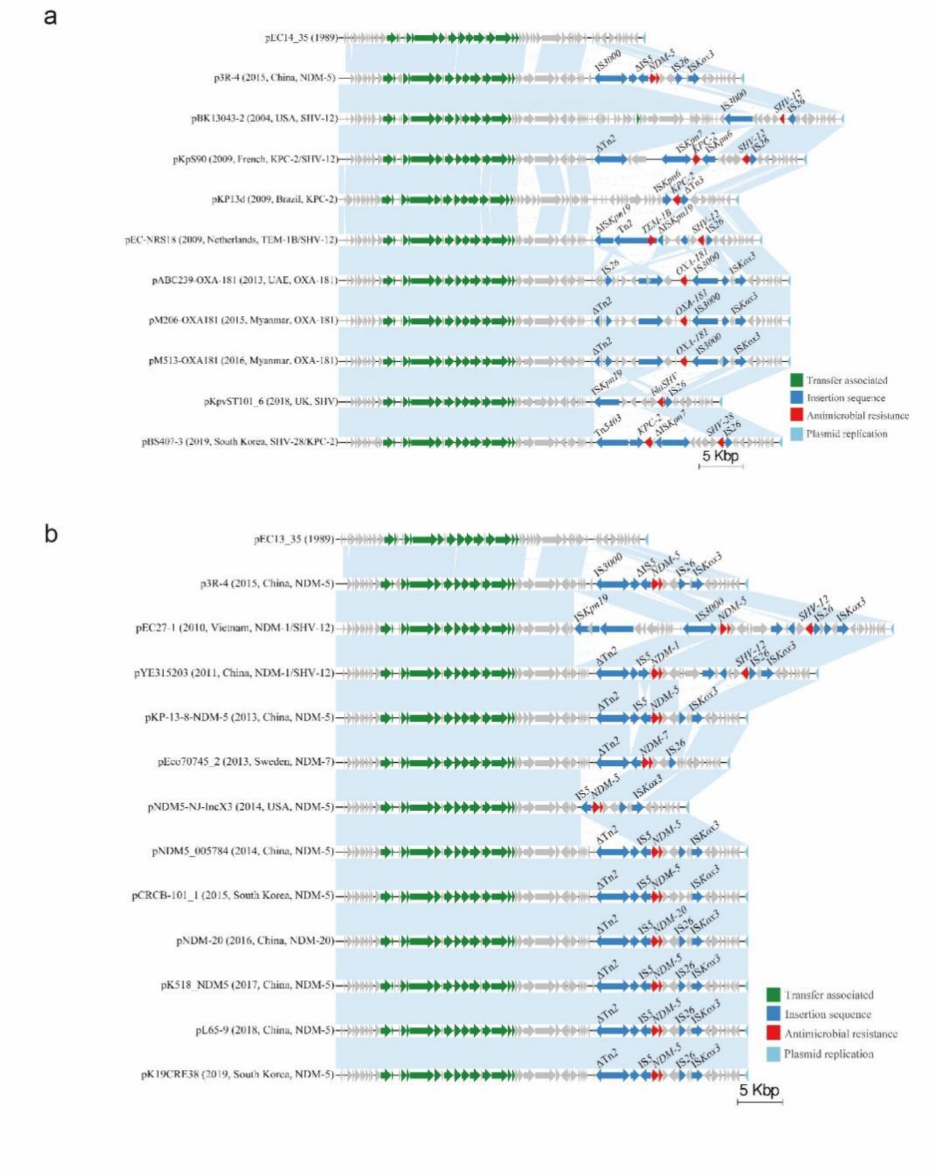

Fig. S3 Sequence alignment of IncX3 plasmids in NCBI database. a. The sequence of alignment of IncX3 plasmid carrying different resistance genes. **b.** The sequence of alignment of IncX3 plasmid carrying different *bla*$_{NDM}$ resistance genes. The light blue shade indicates the homolog regions. Arrows in diverse colors indicates genes of different functions. The name, isolation date, isolation region and resistance gene are listed in the left.

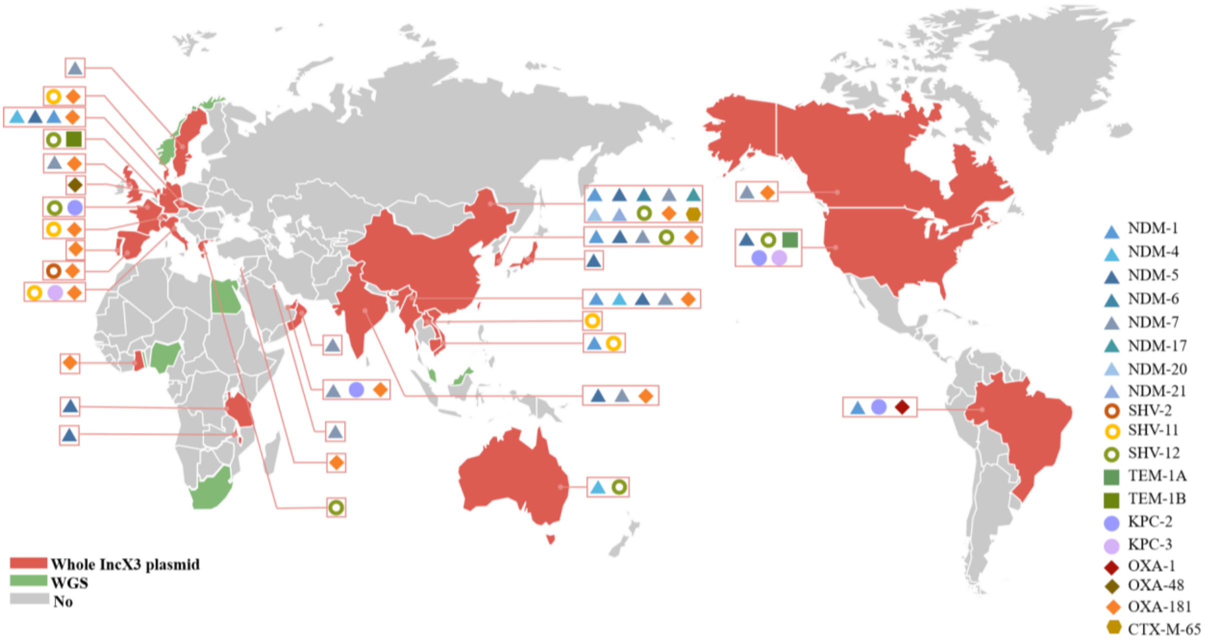

Fig. S4 Worldwide distribution of IncX3 plasmid. The map of the country marked in red indicates that the country has separated and uploaded Whole IncX3 plasmid sequences. The map of the country marked in green indicates that the country has separated and uploaded the whole genome sequence containing the IncX3 plasmid. Grey country maps indicate that no sequences containing IncX3 plasmids have been uploaded for that country.

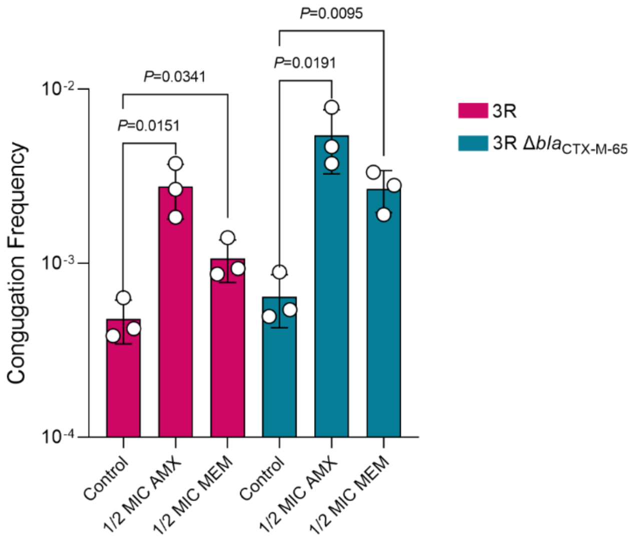

Fig. S5 The in vitro conjugation transfer efficiency of 3R (red bar) and 3RΔ*bla*$_{CTX-M-65}$ (blue bar) strains carrying the IncX3 plasmid under the pressure of either meropenem or amoxicillin. Data are means ± SEM; n = 3 biologically independent replicates. Groups were compared using two-tailed t-test.

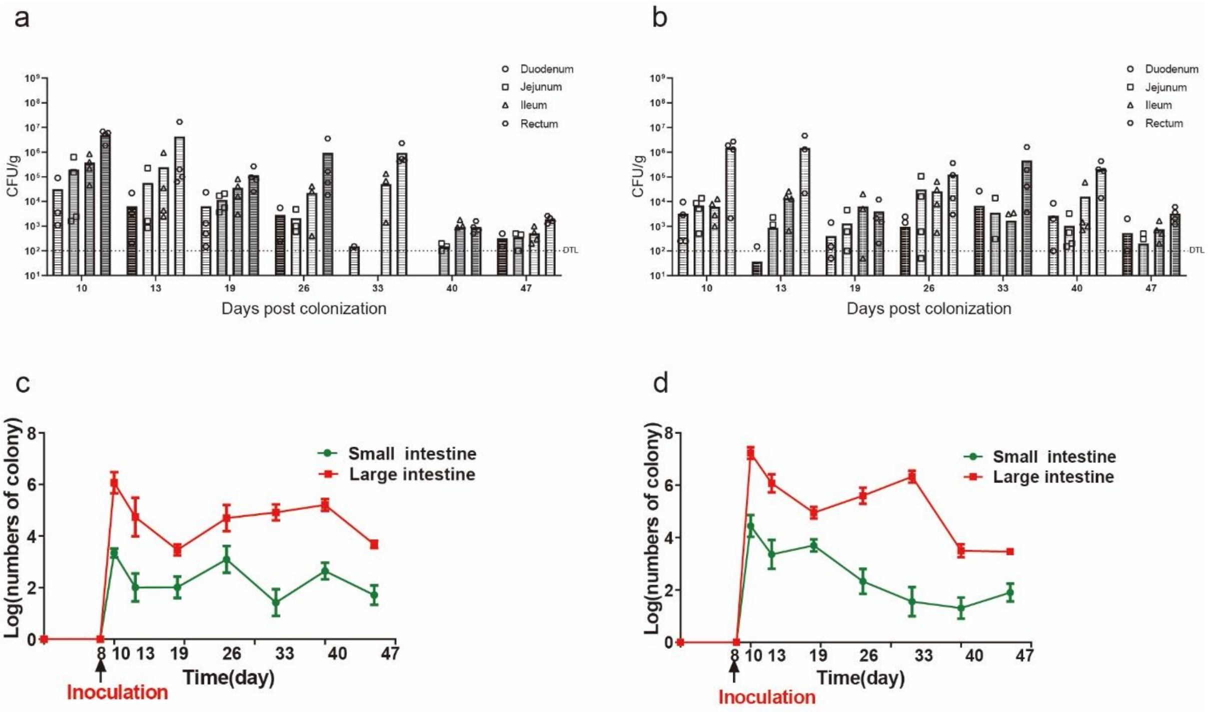

Fig. S6 *E. coli* carrying *bla*$_{NDM-5}$-IncX3 plasmids can persist in chicken intestines. **a.** Bacterial loads of 5 different intestines of inoculated 3R group. **b.** Bacterial loads of 5 different intestines of inoculated 8R group. **c.** *bla*$_{NDM-5}$-positive strains load of large (red line) and small (green line) intestines of inoculated 8R group. **d.** *bla*$_{NDM-5}$-positive strains load of large (red line) and small (green line) intestines of inoculated 3R group.

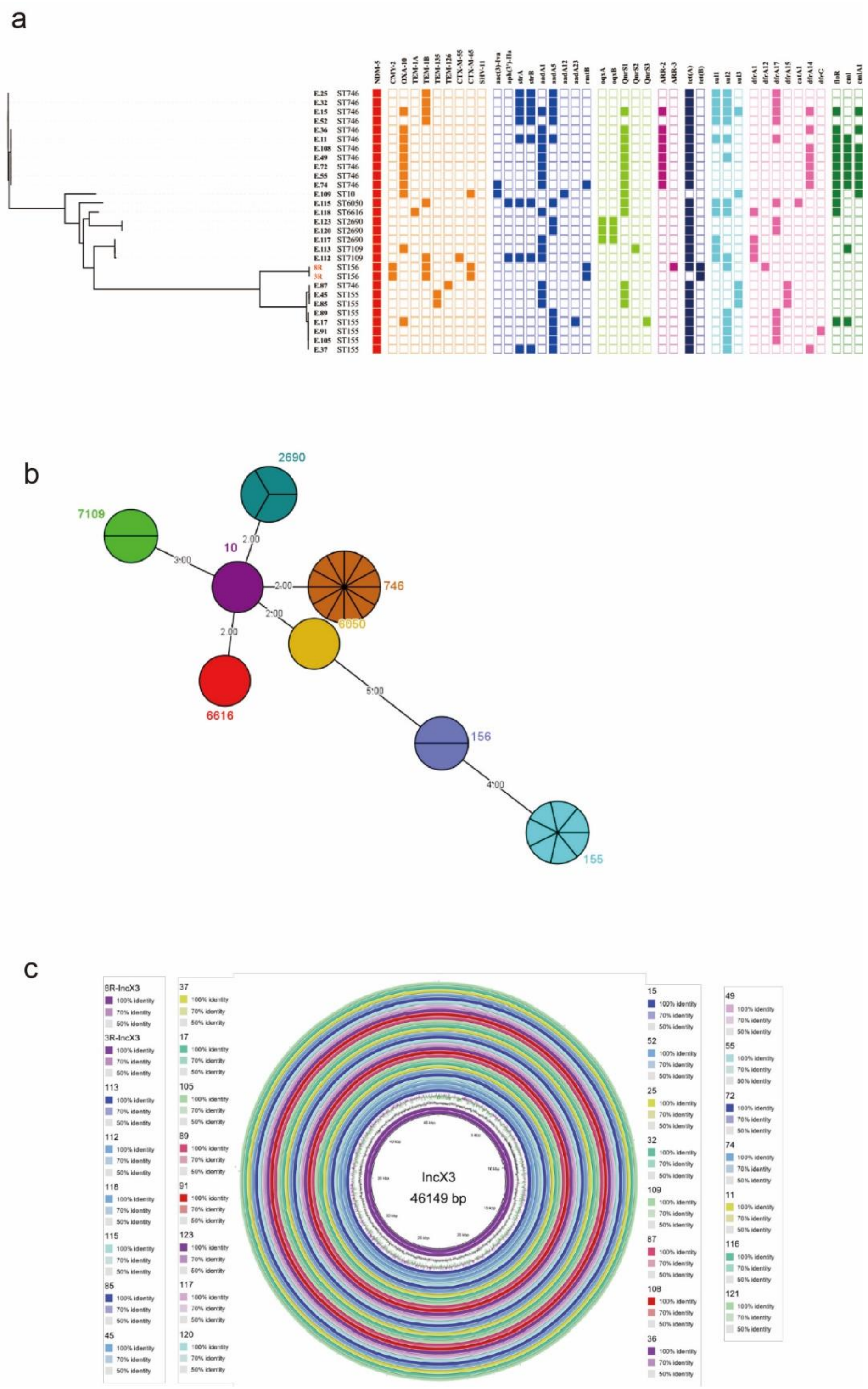

Fig. S7 Core genome-based phylogenetic trees and MLST typing of the Non-inoculated NDM-positive *E. coli* strains. a. Core genome-based phylogenetic trees of the Non-inoculated NDM-positive *E. coli* strains. **b.** MLST typing of the Non-inoculated NDM-positive *E. coli* strains. **c.** The BRIG IncX3 plasmid comparative analysis of Non-inoculated NDM-positive *E. coli* strains.

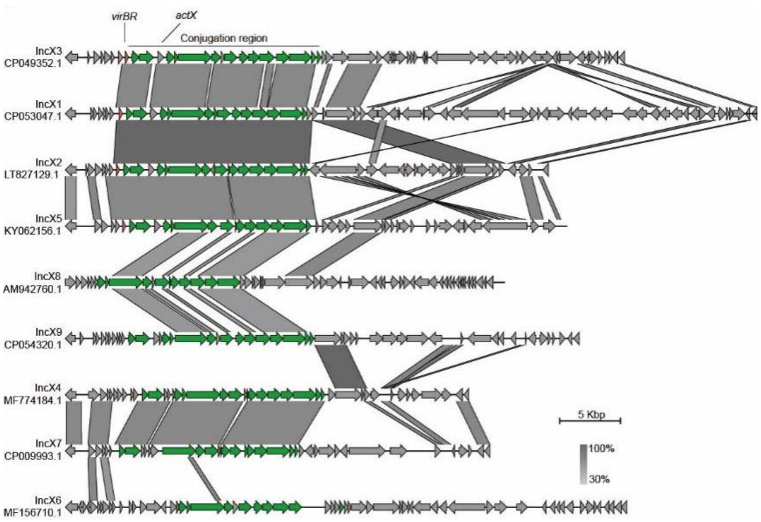

Fig. S8 The multiple sequence alignment of T4SS in IncX1-9. Multiple sequence alignment of T4SS in IncX1-9. Green arrows represent T4SS and red arrow represent *virBR*. The gray values represent the homology. The Genbank number is listed in the left.

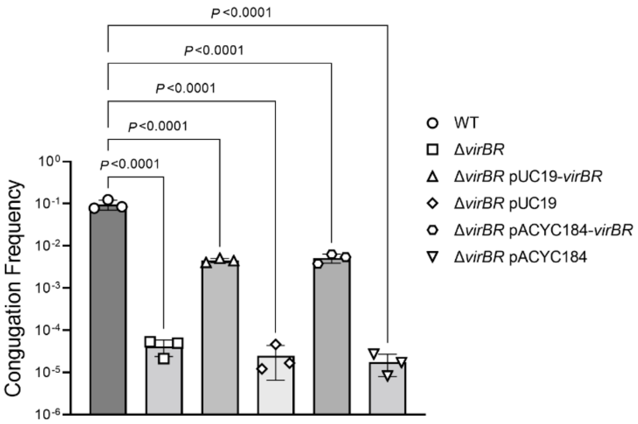

Fig. S9 Conjugation frequency of IncX3 plasmids and its derivatives in *E. coli* BW25113. Data are means ± SEM; n = 3 biologically independent replicates. One-way ANOVA was performed on values.

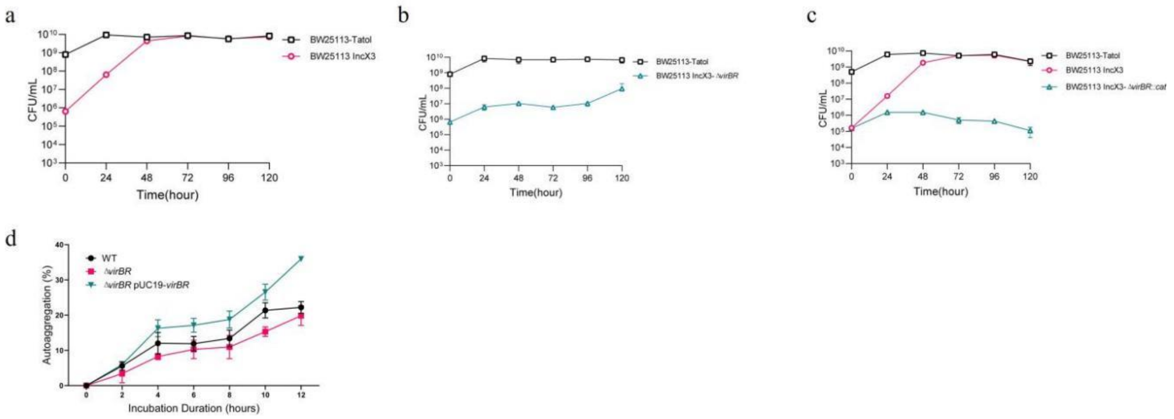

Fig. S10. a. The invasion ability of intact IncX3 plasmid to BW25113 strain. **b.** The invasion ability of IncX3-Δ*virBR* plasmid to BW25113 strain. **c.** Comparison of invasion ability of intact IncX3 plasmid and IncX3-Δ*virBR* plasmid to BW25113 strain. **d.** The autoagglutination ability of 3R, 3R-Δ*virBR*, 3R-Δ*virBR*-pUC19-*virBR* strains.

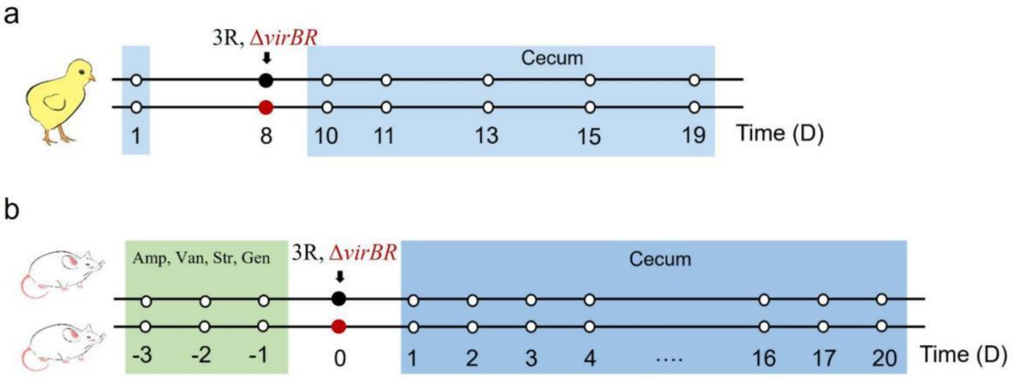

Fig. S11. a. Outline of chicken *in-vivo* experimental design showing strain inoculum (3R, black circle; 3R-Δ*virBR*, red circle) and cecum sampling times (days). Blue box indicates the sample collection date. **b.** Outline of mice *in-vivo* experimental design showing strain inoculum (3R, black circle; 3R-Δ*virBR*, red circle) and cecum sampling times (days). Green box indicates antibiotic challenge prior to challenge by 3R and 3R-Δ*virBR*. Blue box indicates the sample collection date.

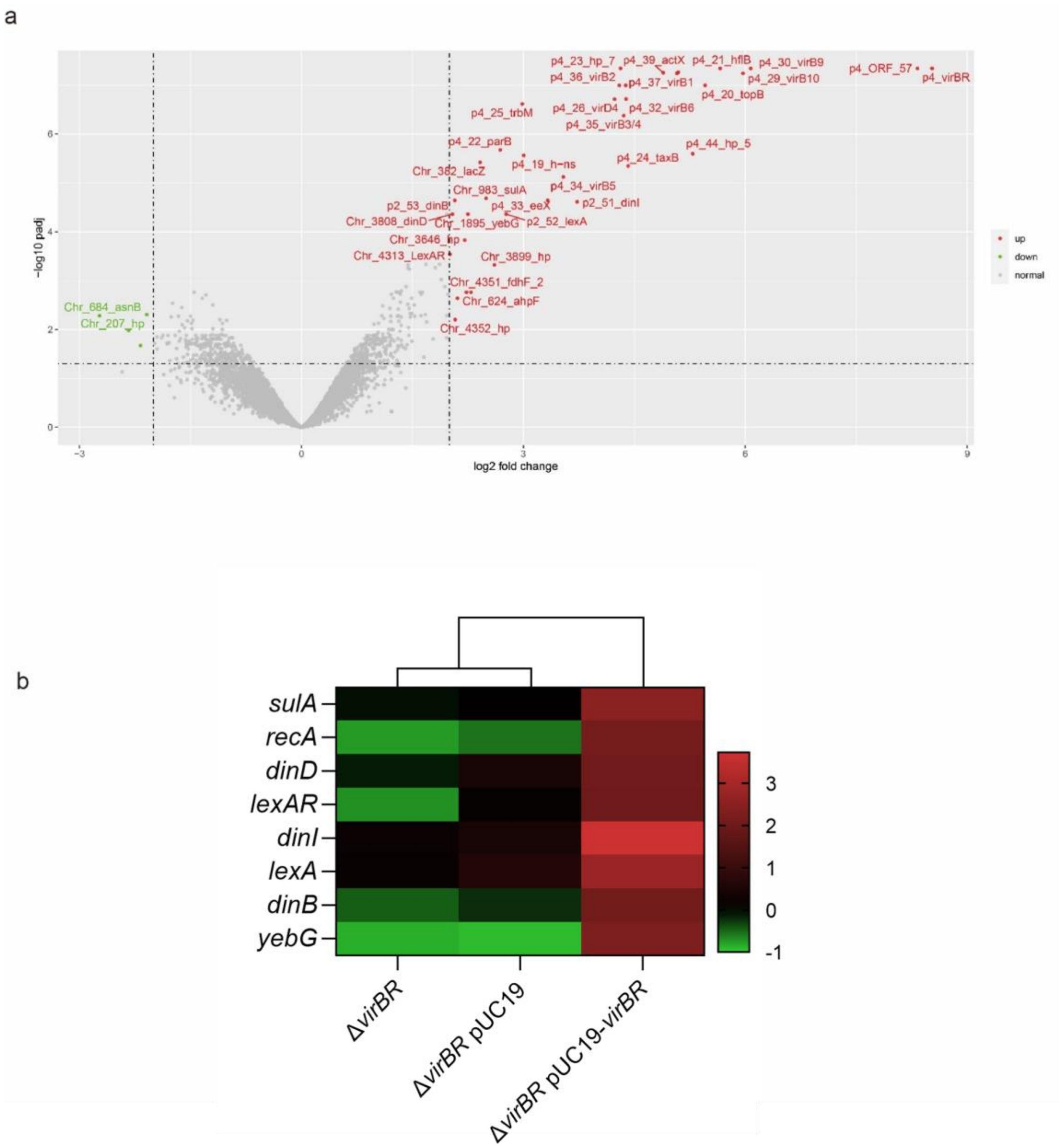

Fig. S12 Fold changes of expression of genes in the condition of overexpression or deletion of *virBR*. a. Fold changes of expression of all genes in the condition of overexpression of *virBR*. Red plot represents up-expression of genes, and green plot represents down-expression of genes. **b**. Fold changes of expression of genes related to SOS response in the condition of overexpression or deletion of *virBR*.

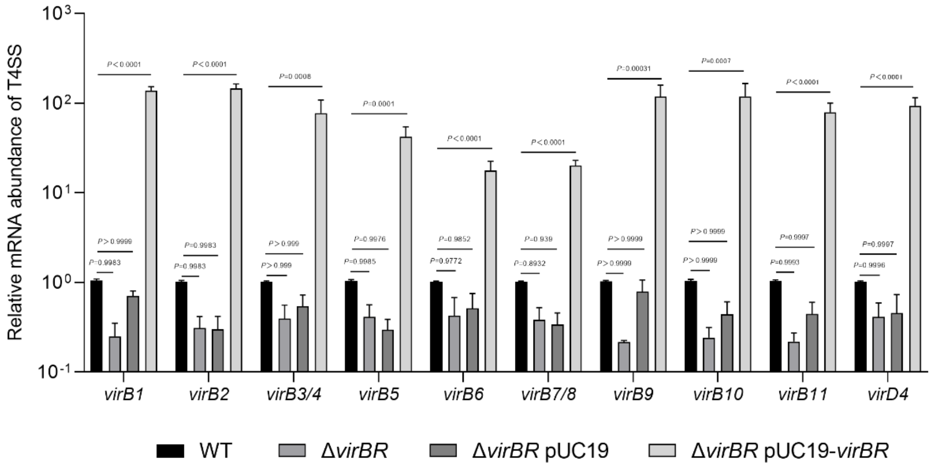

Fig. S13 mRNA levels of T4SS genes in 3R and its derivatives. Data are means ± SEM; n = 3 biologically independent replicates. One-way ANOVA was performed on values.

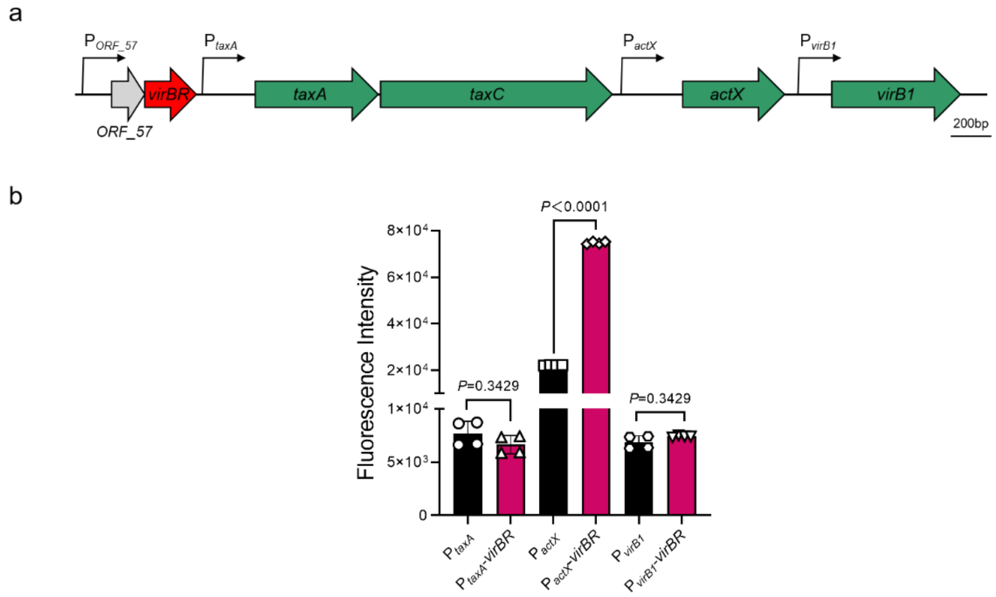

Fig. S14 Action site of *virBR* in the relaxsome and T4SS gene cluster. a. Schematic representation of the putative promoters in the relaxsome and T4SS gene cluster. Red arrows represent *virBR*, and green arrows represent the *virBR* downstream genes. **b**. Fluorescence intensity of GFP under the control of putative promoters in the absence (black bar) or presence (red bar) of *virBR*. Data are means ± SEM; n = 4 biologically independent replicates. Groups were compared using two-tailed t-test.

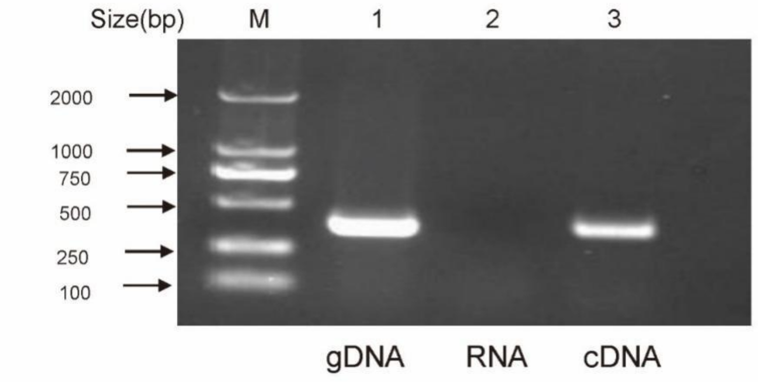

Fig. S15 Co-transcription tests of *actX* and *virB1*. Primers located in the *actX* and *virB1* coding sequence were used to amplified the Genomic DNA (1), RNA (2) and cDNA (3) were used as positive and negative controls respectively.

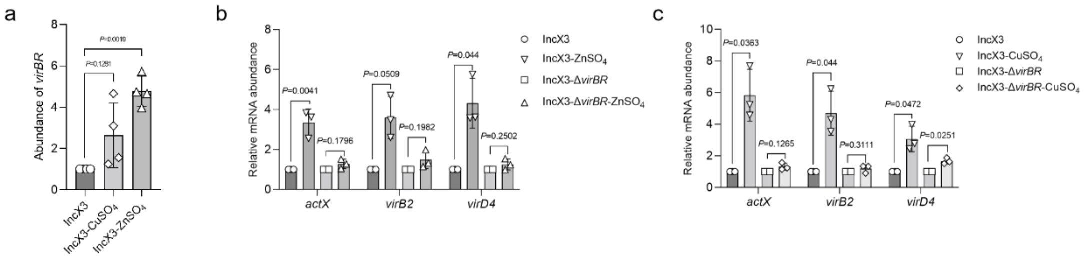

Fig. S16 The mRNA abundance of *virBR* and T4SS genes. **a**. mRNA abundance of *virBR* gene in BW25113-IncX3 in the presence or absence of $ZnSO_4$ or $CuSO_4$. Data are means ± SEM; n = 4 biologically independent replicates. Groups were compared using two-tailed t-test. **b** and **c**. mRNA abundance of partial T4SS genes (*virB2* and *virD4*) in BW25113-IncX3 or BW25113-IncX3-Δ*virBR* in the presence or absence of $ZnSO_4$ or $CuSO_4$. Data are means ± SEM; n = 3 biologically independent replicates. Groups were compared using two-tailed t-test.

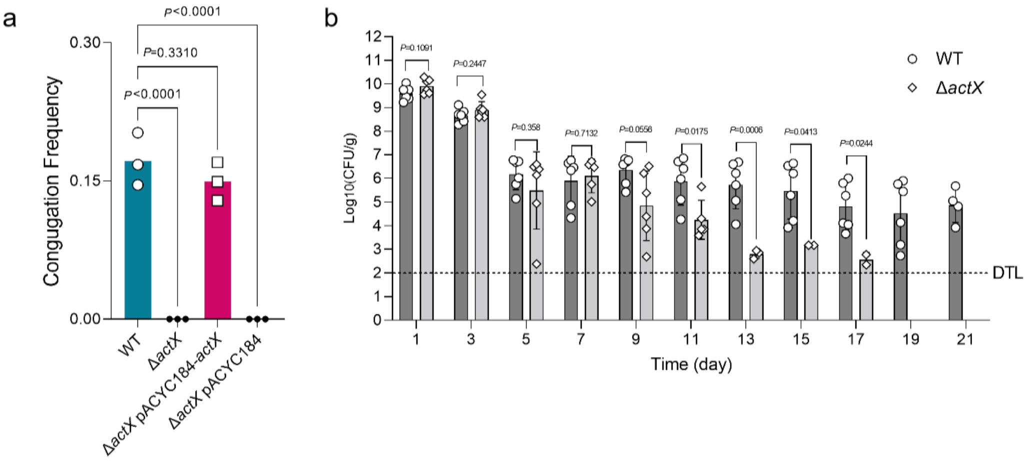

Fig. S17 Strains load of feces of 3R and 3R-Δ*actX* group. a. The conjugation transfer of IncX3 plasmid and its derivatives Δ*actX*. Data are means ± SEM; n = 3 biologically independent replicates. One-way ANOVA was performed on values. **b.** Six mice of each group were euthanized and feces were taken to quantify the inoculated strains at 1, 3, 5, 7, 9, 11, 13, 15, 17, 19, 21 after inoculated. Feces were coated Eosin-Methy Blue Agar Medium (containing 0.25 μg/ml meropenem) to count colony to do inoculated-strains quantitation. Data are means ± SEM; n = 6 biologically independent replicates. Groups were compared using two-tailed t-test.

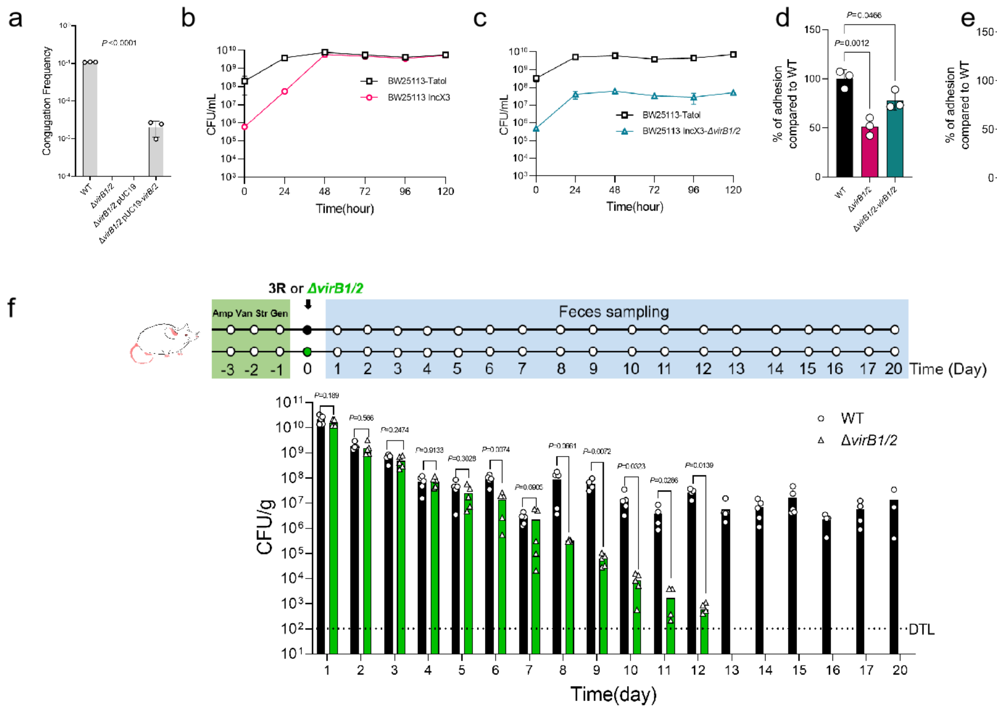

Fig. S18 Effects of the deletion of *virB1/2* on plasmid conjugation, plasmid invasion ability, cell adhesion ability and colonisation. a. The plasmid conjugation ability of 3R and its derivatives. Data are means ± SEM; n = 3 biologically independent replicates. One-way ANOVA was performed on values. **b** and **c**. Plasmid invasion ability of IncX3 plasmid in the presence or absence of *virB1/2*. d and e. The cell adhesion ability of 3R and its derivatives. Data are means ± SEM; n = 3 biologically independent replicates. Groups were compared using two-tailed t-test. f. the colonisation ability of 3R and its derivatives. Black represent WT group, green represent delete of *virB1/2* group. Data are means ± SEM; n = 5 biologically independent replicates. Groups were compared using two-tailed t-test.

References

[1] Wang Y*, et al.* Comprehensive resistome analysis reveals the prevalence of NDM and MCR-1 in Chinese poultry production. *Nature Microbiology* **2**, 16260 (2017).

[2] Wanner KADaBL. One-step inactivation of chromosomal genes in *Escherichia coli* K-12 using PCR products. *PROC NATL ACAD SCI U S A* **97**, (2000).

[3] Matsumura Y, Peirano G, Pitout JDD. Complete Genome Sequence of *Escherichia coli* J53, an Azide-Resistant Laboratory Strain Used for Conjugation Experiments. *Genome Announcements* 6, (2018).

Fig. S15 source data:

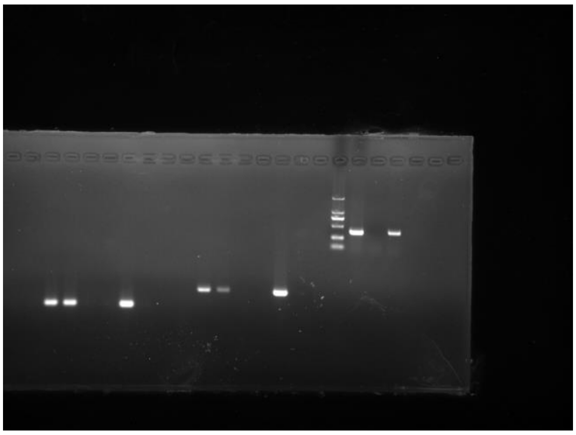

Supplement: Supplementary file 1 — Supplementary Information [file 41467_2024_49800_MOESM1_ESM.pdf]
