# Supplementary material for: VirBR, a transcription regulator, promotes IncX3 plasmid transmission, and persistence of blaNDM-5 in zoonotic bacteria
Source: Nat Commun. 2024 Jun 28;15:5498. doi: 10.1038/s41467-024-49800-1 (PMC11214620; doi:10.1038/s41467-024-49800-1)
Supplement: Supplementary file 3 — Reporting Summary [file 41467_2024_49800_MOESM3_ESM.pdf]

nature portfolio

Corresponding author(s): Yang Wang

Last updated by author(s): Mar 4, 2024

Reporting Summary

Nature Portfolio wishes to improve the reproducibility of the work that we publish. This form provides structure for consistency and transparency in reporting. For further information on Nature Portfolio policies, see our Editorial Policies and the Editorial Policy Checklist.

Statistics

For all statistical analyses, confirm that the following items are present in the figure legend, table legend, main text, or Methods section.

n/a	Confirmed	
☐	☒	The exact sample size (*n*) for each experimental group/condition, given as a discrete number and unit of measurement
☐	☒	A statement on whether measurements were taken from distinct samples or whether the same sample was measured repeatedly
☐	☒	The statistical test(s) used AND whether they are one- or two-sided<br>*Only common tests should be described solely by name; describe more complex techniques in the Methods section.*
☐	☒	A description of all covariates tested
☒	☐	A description of any assumptions or corrections, such as tests of normality and adjustment for multiple comparisons
☐	☒	A full description of the statistical parameters including central tendency (e.g. means) or other basic estimates (e.g. regression coefficient) AND variation (e.g. standard deviation) or associated estimates of uncertainty (e.g. confidence intervals)
☐	☒	For null hypothesis testing, the test statistic (e.g. *F*, *t*, *r*) with confidence intervals, effect sizes, degrees of freedom and *P* value noted<br>*Give P values as exact values whenever suitable.*
☒	☐	For Bayesian analysis, information on the choice of priors and Markov chain Monte Carlo settings
☒	☐	For hierarchical and complex designs, identification of the appropriate level for tests and full reporting of outcomes
☒	☐	Estimates of effect sizes (e.g. Cohen's *d*, Pearson's *r*), indicating how they were calculated

Our web collection on statistics for biologists contains articles on many of the points above.

Software and code

Policy information about availability of computer code

Data collection	TEM images were performed using Electron Microscopy Facility in Oxford University. Fluorescence intensity and OD600 nm values were collected by Tecan Infinite M200 Microplate reader. The Ct values of RT-PCR were performed using ABI quantstudioTM 7 detection system.
Data analysis	GraphPad Prism software (version 9.3.1) or R (version 4.1.2) was used for statistical analysis. Snap Gene (version 5.2) and Easyfig (version2.2.5) were used to analysis the plasmid sequence. CLUSTALW (version 1.2.2) and ESPript (verision 3.0)were used to align the sequence.

For manuscripts utilizing custom algorithms or software that are central to the research but not yet described in published literature, software must be made available to editors and reviewers. We strongly encourage code deposition in a community repository (e.g. GitHub). See the Nature Portfolio guidelines for submitting code & software for further information.

Data

Policy information about availability of data

All manuscripts must include a data availability statement. This statement should provide the following information, where applicable:

- Accession codes, unique identifiers, or web links for publicly available datasets
- A description of any restrictions on data availability
- For clinical datasets or third party data, please ensure that the statement adheres to our policy

All source data in this study were provided in supplementary information. The genome sequencing data of 3R were deposited in GenBank and are registered under

Accession No. SAMN14134968 (https://www.ncbi.nlm.nih.gov/nuccore/SAMN14134968). The genome sequencing data of 8R were deposited in GenBank and are registered under Accession No. CP110410-CP110414 (https://www.ncbi.nlm.nih.gov/nuccore/CP110410.1; https://www.ncbi.nlm.nih.gov/nuccore/CP110411.1; https://www.ncbi.nlm.nih.gov/nuccore/CP110412.1; https://www.ncbi.nlm.nih.gov/nuccore/CP110413.1; https://www.ncbi.nlm.nih.gov/nuccore/CP110414.1). The RAN-sequencing raw data reported in this paper have been deposited in the Genome Sequence Archive (Genomics, Proteomics & Bioinformatics 2021) in National Genomics Data Center (Nucleic Acids Res 2022), China National Center for Bioinformation / Beijing Institute of Genomics, Chinese Academy of Sciences (GSA: CRA015156) that are publicly accessible at https://ngdc.cncb.ac.cn/gsa/browse/CRA015156. Source data are provided with this paper.

Research involving human participants, their data, or biological material

Policy information about studies with human participants or human data. See also policy information about sex, gender (identity/presentation), and sexual orientation and race, ethnicity and racism.

Reporting on sex and gender	*Use the terms sex (biological attribute) and gender (shaped by social and cultural circumstances) carefully in order to avoid confusing both terms. Indicate if findings apply to only one sex or gender; describe whether sex and gender were considered in study design; whether sex and/or gender was determined based on self-reporting or assigned and methods used. Provide in the source data disaggregated sex and gender data, where this information has been collected, and if consent has been obtained for sharing of individual-level data; provide overall numbers in this Reporting Summary. Please state if this information has not been collected. Report sex- and gender-based analyses where performed, justify reasons for lack of sex- and gender-based analysis.*
Reporting on race, ethnicity, or other socially relevant groupings	*Please specify the socially constructed or socially relevant categorization variable(s) used in your manuscript and explain why they were used. Please note that such variables should not be used as proxies for other socially constructed/relevant variables (for example, race or ethnicity should not be used as a proxy for socioeconomic status). Provide clear definitions of the relevant terms used, how they were provided (by the participants/respondents, the researchers, or third parties), and the method(s) used to classify people into the different categories (e.g. self-report, census or administrative data, social media data, etc.) Please provide details about how you controlled for confounding variables in your analyses.*
Population characteristics	*Describe the covariate-relevant population characteristics of the human research participants (e.g. age, genotypic information, past and current diagnosis and treatment categories). If you filled out the behavioural & social sciences study design questions and have nothing to add here, write "See above."*
Recruitment	*Describe how participants were recruited. Outline any potential self-selection bias or other biases that may be present and how these are likely to impact results.*
Ethics oversight	*Identify the organization(s) that approved the study protocol.*

Note that full information on the approval of the study protocol must also be provided in the manuscript.

Field-specific reporting

Please select the one below that is the best fit for your research. If you are not sure, read the appropriate sections before making your selection.

☒ Life sciences ☐ Behavioural & social sciences ☐ Ecological, evolutionary & environmental sciences

For a reference copy of the document with all sections, see nature.com/documents/nr-reporting-summary-flat.pdf

Life sciences study design

All studies must disclose on these points even when the disclosure is negative.

Sample size	Sample sizes were not predetermined based on statistical methods, but were chosen according to the standards in the field - at least 3 independent biological replicates for each condition. This generated sufficient data for statistical analysis.
Data exclusions	No data were excluded from the analysis.
Replication	All results in this study were reproducible in three independent biological.
Randomization	For animal studies, ROSS chickens and mice were randomly assigned to experimental and the control groups.
Blinding	Investigators were not blinded.

Reporting for specific materials, systems and methods

We require information from authors about some types of materials, experimental systems and methods used in many studies. Here, indicate whether each material, system or method listed is relevant to your study. If you are not sure if a list item applies to your research, read the appropriate section before selecting a response.

Materials & experimental systems

n/a	Involved in the study	
☒	☐	Antibodies
☐	☒	Eukaryotic cell lines
☒	☐	Palaeontology and archaeology
☐	☒	Animals and other organisms
☒	☐	Clinical data
☒	☐	Dual use research of concern
☒	☐	Plants

Methods

n/a	Involved in the study	
☒	☐	ChIP-seq
☒	☐	Flow cytometry
☒	☐	MRI-based neuroimaging

Eukaryotic cell lines

Policy information about cell lines and Sex and Gender in Research

Cell line source(s)	Caco-2 cells from Kunming Cell Bank of Chinese Academy of Sciences (KCB200710YJ) and Rat small intestine cell IEC-6 from Shanghai Fuxiang Biotechnology Co., LTD (ATCC CRL-1592)
Authentication	Caco-2 and IEC-6 cell were confirmed by using morphologic observations and species verification by isoenzymology, STR and other quality control tests from supplier.
Mycoplasma contamination	PCR-test was used to detect the mycoplasma. No cells were contaminated with mycoplasma throughout the experiment.
Commonly misidentified lines (See ICLAC register)	No commonly misidentified cell lines were used in the experiment.

Animals and other research organisms

Policy information about studies involving animals; ARRIVE guidelines recommended for reporting animal research, and Sex and Gender in Research

Laboratory animals	six to eight-week-old female mice and one-day-old ROSS chickens
Wild animals	none
Reporting on sex	none
Field-collected samples	The animals were housed in sterile isolators at the Laboratory Animal Public Service Platform of China Agricultural University with antibiotic-free feed and water.
Ethics oversight	The chickens and mice were raised and handled in compliance with the Chinese laws and guidelines (protocol GKFCZ2001545), EU Directive 2010/63/EU for animal experiments, and the China Agricultural University regulations concerning the protection of animals used for scientific purposes (2010-SYXK-0037). Committee on Animal Welfare and Ethics in China Agricultural University approved the chickens and mice experiments (AW32803202-2-1 and AW32803202-2-2).

Note that full information on the approval of the study protocol must also be provided in the manuscript.

Plants

Seed stocks	none
Novel plant genotypes	none
Authentication	none
